# Boundary distinction interpretation of microarray data via discrete correlate summation

**DOI:** 10.1186/gb-2011-12-s1-p27

**Published:** 2011-09-19

**Authors:** Brian M Westwood, Amy L Olex, James L Norris, Leslie B Poole, Jacquelyn S Fetrow

**Affiliations:** 1 Department of Molecular Genetics and Genomics, Wake Forest University School of Medicine, Winston-Salem, NC 27157, USA; 2 Department of Computer Science, Wake Forest University, Winston-Salem, NC 27109, USA; 3 Department of Biochemistry, Wake Forest University School of Medicine, Winston-Salem, NC, 27157, USA; 4 Department of Physics, Wake Forest University, Winston-Salem, NC 27109, USA

## Background

Given differential gene expression data across divergent mutant strain arrays of two enzyme subgroups, it would be logical to segregate by protein group ablation (PGA). Discrete correlate summation (DCΣ) was utilized to examine the differential effects of a hydrogen peroxide stressor on discrete and total yeast knockouts of the genes encoding glutathione peroxidase (Gpx) and peroxiredoxin (Prx), both groups starting from the wild-type (WT) strain [1]. While the half-life of the total Gpx knockout mutant is intermediate between that of the WT and the transient total Prx knockout mutant, the distribution of passage number of the various mutant strains can be separated into two groups independent of Gpx and Prx state. Based on half-viability, totalPrx <<<< nPrx << Gpx3 = Tsa1 < totalGpx < mPrx <<< Gpx1 < Gpx2 << Ahp1 = WT <<< Tsa2 (*P* < 0.0005, two tailed *t*-test, *n* = 5, 6). DCΣ was also employed for the boundary between robust and gracile cultures. The aim of this study was to find the characteristic response of the transcriptome, from the perspective of PGA versus strain viability (SV).

## Methods

DCΣ is a method used to score variables that can be classified into two groups [2]. It is a composite score of a gene’s mean group change and overall interaction difference relative to all others tested. Transcripts were included in this analysis only if the values for all conditions passed microarray quality control and were present in the Kyoto Encyclopedia of Genes and Genomes (KEGG) network [3]. Randomly sorted edges were sampled for comparison (*P* < 0.001, two tailed *t*-test, *n* = 8,372). Edges that were sorted on average DCΣ score and grouped by biological process yielded a distinctive topology (*P* < 1e–85, two tailed *t*-test, *n* = 8,372). The identified transcripts were subjected to functional annotation in the Database for Annotation, Visualization and Integrated Discovery (DAVID) [4].

## Results

Application of DCΣ to the individual and complete knockouts of Gpx (3 genes) and Prx (5 genes) identified 92 transcripts based on PGA and 43 based on SV, with a 13 gene overlap (corresponding to the proteins Arg1p, Aah1p, Ade17p, Pgm2p, Cat2p, Cdd1p, Mae1p, Arg3p, Nma2p, Ole1p, Cta1p, Spb1p and Cds1p). Functional annotation analysis of the 92 PGA transcripts identified the following functions: pyrimidine metabolism, steroid biosynthesis, purine metabolism, RNA polymerase and terpenoid backbone biosynthesis. Ergosterol biosynthesis, gluconeogenesis and transcription from Pol I/III promoters were major biological process categories for this set. Interestingly, terpenoids feed into the steroid pathway, which results in the vitamin D2 precursor ergosterol. Analysis of the 43 SV transcripts identified starch and sucrose metabolism, butanoate metabolism, and fructose and mannose metabolism. Stress response was the key biological process for this arm of the study. No functional annotations were statistically significant for the common genes. Transcripts identified by PGA of either the Gpx- or Prx-encoding genes tend toward transcriptional control mechanisms, whereas SV-associated transcripts track with metabolic necessities.
